# Impact of lenalidomide maintenance on the immune environment of multiple myeloma patients with low tumor burden after autologous stem cell transplantation

**DOI:** 10.18632/oncotarget.24944

**Published:** 2018-04-17

**Authors:** Karel Fostier, Jo Caers, Nathalie Meuleman, Katrijn Broos, Jurgen Corthals, Kris Thielemans, Rik Schots, Brenda De Keersmaecker

**Affiliations:** ^1^ Vrije Universiteit Brussel (VUB), Universitair Ziekenhuis Brussel (UZ Brussel), Department of Hematology, Brussels, Belgium; ^2^ Centre Hospitalier Universitaire (CHU) de Liège, Department of Hematology, Liège, Belgium; ^3^ Institut Jules Bordet, Department of Hematology, Brussels, Belgium; ^4^ Vrije Universiteit Brussel (VUB), Laboratory of Molecular and Cellular Therapy, Brussels, Belgium

**Keywords:** lenalidomide, immunomodulation

## Abstract

Lenalidomide is a potent anti-myeloma drug with immunomodulatory properties. It is increasingly used in a low-dose maintenance setting to prolong remission duration after standard treatment. Data on the *in vivo* effects of lenalidomide are scarce and sometimes different from the well-described *in vitro* effects. We therefore evaluated the numerical, phenotypical and functional impact of lenalidomide maintenance on several immune cell types in a cohort of seventeen homogeneously treated myeloma patients achieving a low residual myeloma burden after a bortezomib based-induction followed by autologous stem cell transplantation. Lenalidomide maintenance: 1) increased the fraction of naïve CD8^+^ T cells and several memory T-cell subsets, 2) reduced the numbers of terminal effector CD8^+^ T cells, 3) resulted in a higher expression of co-stimulatory molecules on resting T cells and of the inhibitory checkpoint molecules LAG-3 on CD4^+^ T cells and TIM-3 on CD4^+^ and CD8^+^ T cells, 4) reduced the number of TIGIT^+^ CD8^+^ T cells, 5) increased the number of regulatory T cells with a phenotype associated with strong suppressive capacity. Purified CD8^+^ T cells showed increased and more polyfunctional recall viral responses. However, PBMC responses were not enhanced during lenalidomide maintenance and CD4^+^ T-cell responses specific for the myeloma-associated antigen MAGE-C1 even tended to become lower. We conclude that lenalidomide maintenance after autologous stem cell transplantation has complex pleotropic effects on the immune environment. Immune interventions such as anti-myeloma vaccination should include measures to tackle an expanded inhibitory Treg compartment.

## INTRODUCTION

Maintenance treatment with the immunomodulatory drug (IMiD) lenalidomide prolongs progression-free and overall survival in multiple myeloma (MM) patients after autologous stem cell transplantation (ASCT). An important mechanism of action of lenalidomide is its effect on the immune system, which is heavily disrupted in MM patients. Various studies have demonstrated the *in vitro* immunomodulatory effects of lenalidomide: increased natural killer (NK) cell cytotoxicity [1], improved functionality of invariant NKT cells [2] and T-cell co-stimulatory capacity [1, 3], resulting in broad and polyfunctional antigen-specific T-cell responses with a high antigen sensitivity [4, 5]. Interestingly, the addition of lenalidomide to T-cell cultures results in a decreased expression of the inhibitory immune checkpoint molecule programmed death protein 1 (PD-1) while potentiating responses to a dendritic cell (DC)/myeloma fusion vaccine [6]. Lenalidomide diminishes the expression of suppressor of cytokine signaling (SOCS)1 on T cells, NK cells and NKT cells from both the bone marrow and the peripheral blood of MM patients [7]. Furthermore, lenalidomide induces the degradation of T cell repressors through modulation of cereblon [8]. Finally, lenalidomide inhibits the proliferation and T-cell suppressive function of regulatory T cells (Tregs) *in vitro* [5, 6, 9].

The *in vivo* effects of lenalidomide treatment on the immune environment are much less documented. A recent study demonstrated that CD4^+^ T cells play a major role in the therapeutic effects of lenalidomide on immunocompetent mice bearing 5TGM1 MM cells. In addition, lenalidomide significantly increased the numbers of IFN-γ^+^ T cells and perforin^+^ CD8^+^ T cells while slightly reducing the numbers of Tregs in this mouse model [10]. Busch e*t al.* compared immune characteristics of MM patients treated with a lenalidomide mono- or combination therapy to these of MM patients treated with other agents. They found that a lenalidomide-containing treatment regimen was associated with higher numbers of CD8^+^ T cells phenotypically staged between memory T cells and effector memory T cells. In addition, lenalidomide-treated patients showed a higher abundance of CD14^+^ CD15^+^ myeloid cells with a T-cell inhibitory capacity (MDSCs) [11]. Clave *et al.* studied the effect of lenalidomide treatment on T-cell immune reconstitution in patients with MM who had undergone ASCT. Lenalidomide impaired long-term thymic T-cell reconstitution, decreased the number of CD4^+^ and CD8^+^ CD45RA^+^ CCR7^-^ terminal effector T cells while increasing the number of Tregs [12]. Lenalidomide induction or maintenance therapy does not affect NKT cell numbers [13]. Recently, Krämer *et al.* compared the immune environment in MM patients treated with or without lenalidomide. They found increased frequencies of CD8^+^ T-cell responses for the MM-associated antigen HM1.24 in patients treated with lenalidomide compared to patients without lenalidomide treatment [14]. Upon PMA/ionomycin stimulation higher numbers of IFN-γ, TNF-α and IL-21 secreting T cells were detected in MM patients under lenalidomide maintenance treatment compared to MM patients that did not receive lenalidomide [15].

Since *in vitro* and *in vivo* studies report conflicting results on certain aspects of immunomodulation mediated through lenalidomide and given the rather limited information currently available on the *in vivo* effects of lenalidomide given as mono-therapy in maintenance treatment, we performed a detailed analysis to further elucidate the effects of this immunomodulating drug on the immune environment in MM patients achieving a low tumor burden after ASCT.

## RESULTS

### Patient predisposition and timepoints

The patient characteristics are summarized in Table 1. Median age at diagnosis was 59.2 years. 5/17 patients had ISS stage 3 and 2/17 had adverse cytogenetics at diagnosis (either a gain of 1q, deletion 17p or translocation (4;14)), leading to 7 high risk patients. All patients received a bortezomib based induction (either bortezomib-dexamethasone (VD) or bortezomib-thalidomide-dexamethasone (vtD)). 4/17 patients received 2 additional cycles of vtD consolidation after ASCT. 10/17 obtained a VGRP, 1/17 a CR and 6/17 obtained a stringent CR after ASCT or consolidation. The pre-LEN timepoint was assessed at a median of 18.1 weeks, LEN was started at a median of 25.6 weeks and the LEN timepoint was assessed at a median of 41.3 weeks, all after ASCT. The median duration of LEN maintenance was 18.0 weeks (range : 8–37.4 weeks).

**Table 1:** Patient characteristics

Patient number	Age	ISS	Sex	High risk cytogenetics	Induction	Conso-lidation	Response post ASCT or consolidation	Pre LEN timepoint (weeks after ASCT)	Start LEN maintenance (weeks after ASCT)	LEN Timepoint (weeks after ASCT)
1	63,2	2	F	NO	VD × 4	NO	sCR	25,9	28,0	62,0
2	59,2	1	F	NO	vtD × 4	NO	VGPR	22,4	25,6	54,6
3	63,0	1	F	NO	vtD × 4	NO	VGPR	35,4	38,1	48,6
4	51,7	2	F	YES (gain 1q)	VD × 4	NO	VGPR	16,4	17,6	29,6
5	60,0	2	F	NO	VD × 4	NO	sCR	18,9	24,0	41,0
6	63,6	2	M	YES (t(4;14) and del(17p))	vtD × 4	NO	VGPR	15,9	29,0	47,0
7	62,7	1	M	NA	vtD × 4	NO	sCR	15,9	20,7	41,0
8	64,7	3	M	NA	vtD × 4	vtD × 2	VGPR	23,9	26,9	57,3
9	66,0	3	M	NO	vtD × 4	vtD×2	CR	25,9	29,6	59,0
10	61,9	3	F	NA	vtD × 4	NO	VGPR	13,1	17,1	39,9
11	52,0	3	M	NO	vtD × 4	vtD x 2	VGPR	12,9	25,0	46,0
12	58,1	2	M	NO	vtD × 4	vtD x 2	VGPR	23,1	26,0	41,3
13	53,7	3	F	NA	vtD × 4	NO	CR	14,1	16,3	24,3
14	59,1	1	F	NO	VD × 5	NO	VGPR	15,4	27,1	64,6
15	58,4	2	M	NO	vtD × 4	NO	sCR	21,9	29,7	40,0
16	55,9	2	F	NO	vtD × 4	NO	sCR	18,1	21,3	35,3
17	51,9	1	M	NO	vtD × 4	NO	VGPR	17,1	25,0	39,3

VD: Bortezomib - Dexamethasone. vtD: Bortezomib-Thalidomide-Dexamethasone. VGPR: Very good partial response. CR: complete response. sCR: stringent complete response. NA: not available. ISS: international staging system. M: Male, F: Female.

### Lenalidomide maintenance therapy results in higher numbers of naïve CD8^+^ T cells and memory T cells while reducing the number of terminal effector T cells

**Figure 1:**
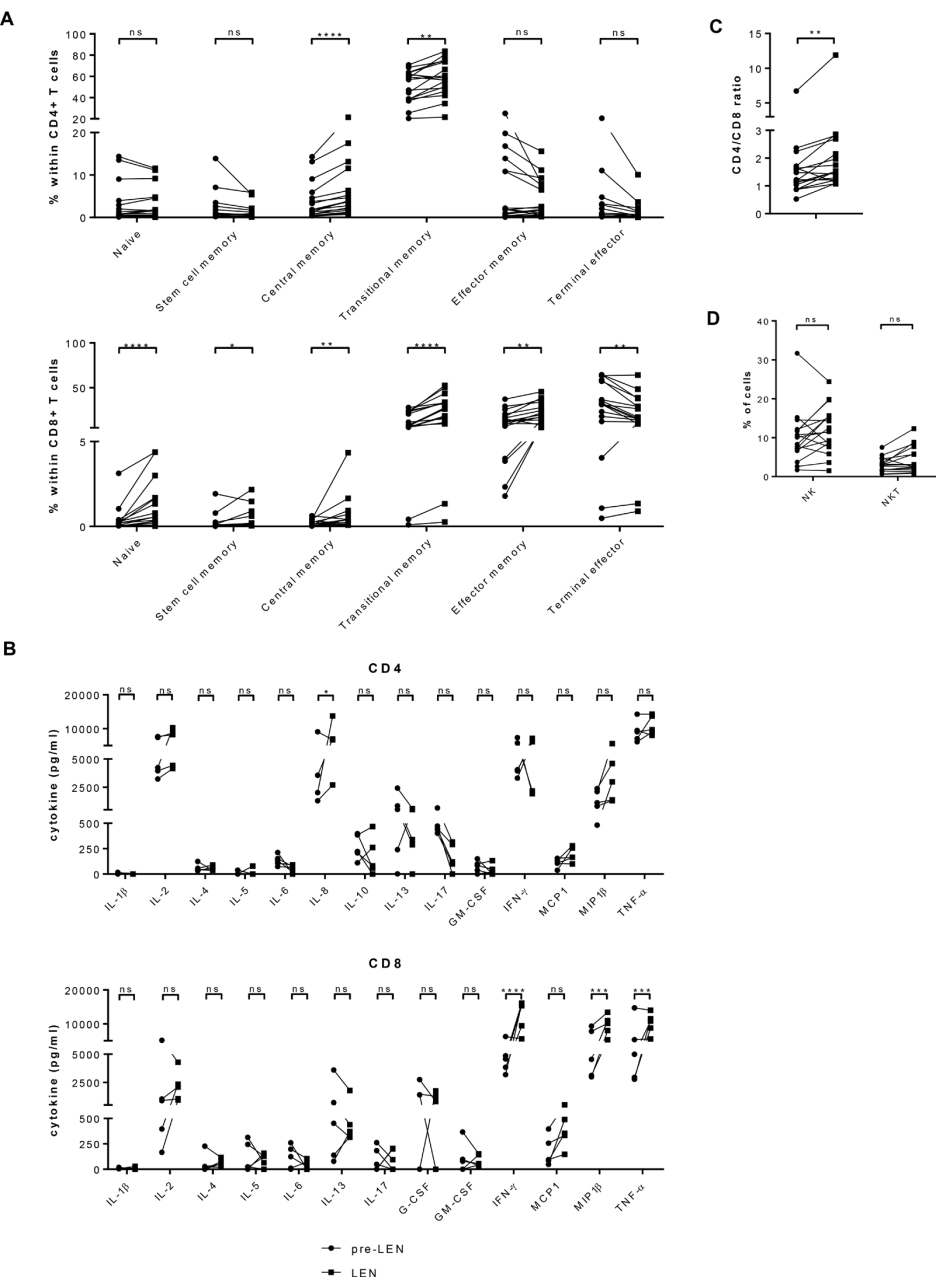
Lenalidomide maintenance treatment results in increased numbers of naïve CD8^+^ T cells and memory T cells while reducing the number of terminal effector T cells PBMCs obtained before (pre LEN) and during (LEN) lenalidomide maintenance treatment were examined by flow cytometry to determine the number of different types of immune effector cells (*n* = 17). (**A**) The proportion of CD4^+^ and CD8^+^ T cells belonging several T cell subsets was determined. Naïve T cells were defined as CD45RO^–^ CCR7^+^ CD28^+^ CD95^–^, stem cell memory T cells were defined as CD45RO^–^ CCR7^+^ CD28^+^ CD95^+^, central memory T cells were defined as CD45RO^+^ CCR7^+^ CD28^+^ CD95^+^, transitional memory T cells were defined as CD45RO^+^ CCR7^–^ CD28^+^ CD95^+^, effector memory T cells were defined as CD45RO^+^ CCR7^–^ CD28^–^ CD95^+^ and terminal effector T cells were defined as CD45RO^–^ CCR7^–^ CD28^–^ CD95^+^ (Cfr. Supplementary Figure 1). (**B**) Cytokine production upon polyclonal stimulation was analyzed using multiplex assay. Cytokine measured upon culture in the absence of stimulus was considered as background and subtracted from cytokine concentrations measured upon stimulation with anti-CD3 and anti-CD28 coated microbeads. Results were analyzed using two-way ANOVA with Sidak’s multiple comparison tests. (**C**) The ratio of CD4^+^/CD8^+^ T cells is depicted on the graph. (**D**) The percentage of NK cells was defined as the percentage of CD3^–^ CD56^+^ CD16^+^ cells within the PBMC population. The percentage of NKT cells was defined as the percentage of CD3^+^ CD56^+^ cells. The Wilcoxon matched-pairs signed rank test was performed to evaluate statistical significant differences, ns: not significant, ^*^p ≤ 0.05, ^**^p ≤ 0.01 and ^****^p ≤ 0.0001.

We evaluated the effects of lenalidomide maintenance treatment on the distribution of CD4^+^ and CD8^+^ T cells over several subtypes (Figure 1A and Supplementary Figure 1). Our flow cytometry staining panel was based on a publication by Mahnke *et al.* [16]. We observed that the percentage of naïve CD8^+^ T cells increased significantly during lenalidomide maintenance (mean: 0.36% versus 1.15%) while the frequency of naïve CD4^+^ T cells remained stable (mean: 2.99% versus 3.03%). The fraction of stem cell memory CD8^+^ T cells showed an increase (mean: 0.22% pre-LEN versus 0.37% LEN) yet stem cell memory CD4^+^ T cells remained stable during the treatment (1.96% pre-LEN versus 1.25% LEN, respectively). The percentages of central memory CD4^+^ and CD8^+^ T cells were both significantly higher during the lenalidomide maintenance therapy (mean: 3.81% versus 6.06% and 0.16% versus 0.61%, respectively). In addition, we observed a highly significant increased number of transitional memory T cells upon lenalidomide treatment (mean: 50.34% versus 57.88% for the CD4^+^ T cells and 13.09% versus 25.31% for the CD8^+^ T cells). Lenalidomide maintenance resulted in lower percentages of effector memory CD4^+^ T cells (mean: 6.39% versus 4.15%), but this difference was not statistically significant. In contrast, within the CD8^+^ T-cell population, the fraction of effector memory T cells did increase significantly during lenalidomide maintenance treatment (from 13.77% to 20.23%, on average). Finally, the percentage of terminal effector T cells decreased in both CD4^+^ and CD8^+^ T-cell compartments upon lenalidomide maintenance and this difference was significant in the CD8^+^ T-cell compartment (mean: from 2.82% to 1.33% and from 33.20% to 22.47%, respectively).

We further investigated the capacity of the T cells to produce cytokines upon activation by performing a multiplex assay. As shown in Figure 1B, the capacity of the CD4^+^ T cells to produce IL-8 was significantly increased during lenalidomide maintenance (mean: 3992 pg/ml versus 7532 pg/ml). The CD8^+^ T cells obtained during lenalidomide maintenance produced higher amounts of IFN-γ (mean: 4550 pg/ml versus 12496 pg/ml) MIP-1β (mean: 5567 pg/ml versus 9583 pg/ml) and TNF-α (mean: 6156 pg/ml versus 10128 pg/ml). For the other tested cytokines, no significant differences between the pre-LEN and the LEN timepoint were detected.

Interestingly, lenalidomide treatment also resulted in a higher CD4^+^/CD8^+^ T cell ratio (mean: 1.66 before and 2.30 during lenalidomide maintenance) (Figure 1C). No significant differences in the frequency of NK cell numbers were observed between both timepoints. We did find higher percentages of NKT cells during lenalidomide maintenance treatment (3.05% NKT cells pre-LEN versus 4.22% NKT cells during LEN) (Figure 1D and Supplementary Figure 2).

### Lenalidomide maintenance therapy has opposite effects on Treg and monocytic MDSC numbers

**Figure 2:**
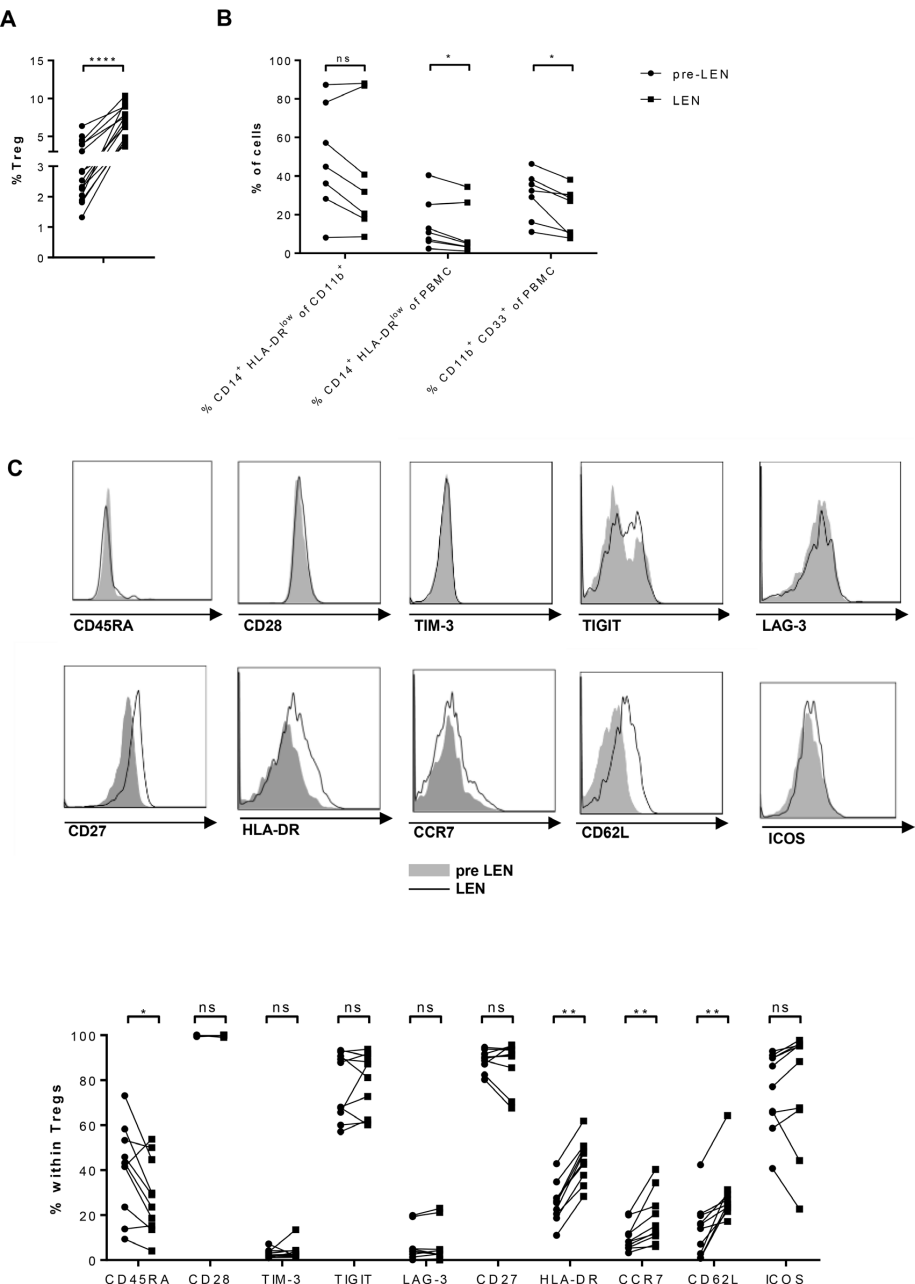
Lenalidomide maintenance therapy has opposite effects on Treg and monocytic MDSC numbers PBMCs obtained before (pre LEN) and during (LEN) lenalidomide maintenance treatment were examined by flow cytometry to determine the number of different types of immune suppressive cells. (**A**) The proportion of Tregs, defined as CD4^+^ CD25^high^ CD127^low^ Foxp3^high^ cells (Cf. Supplementary Figure 3), was determined (*n* = 17). (**B**) The percentage of monocytic MDSC (moMDSC), defined as the percentage of CD14^+^ HLA-DR^low^ cells within either the PBMCs or the CD11b^+^ population as well as the percentage of CD11b^+^ CD33^+^ PBMCs was determined (*n* = 7). (**C**) The phenotype of Tregs was further dissected. The expression of CD45RA, CD28, TIM-3, TIGIT, LAG-3, CD27, GITR, HLA-DR, CCR7, CD62L and ICOS on Tregs was evaluated (*n* = 10). The Wilcoxon matched-pairs signed rank test was performed to evaluate statistical significant differences, ns: not significant, ^*^p ≤ 0.05, ^**^p ≤ 0.01, ^***^p < 0.001 and ^****^p < 0.0001.

Next to the immune effector cells, we evaluated the frequency of some immune suppressive cell types in the patients. We assessed the percentage of Tregs (gating strategy, see Supplementary Figure 3) before and during lenalidomide (Figure 2A). We observed a highly significant increase in the number of Tregs upon lenalidomide maintenance (mean: 3.14% versus 7.91%). Compared to MM patients, healthy donors showed lower Treg numbers (mean: 2.22%) and the difference with MM patients treated with lenalidomide was highly significant (Supplementary Figure 4). We further evaluated the presence of MDSCs in our patients. Since granulocytic cells are not present in the PBMCs we had available for the analysis, we focused on monocytic MDSCs (moMDSCs, gating strategy see Supplementary Figure 3). In contrast to the Tregs, we observed significantly reduced percentages of moMDSCs defined as percentage of CD33^+^ CD11b^+^ CD14^+^ HLA-DR^low^ cells within the PBMC population (Figure 2B) (15.04% versus 11.31%) during lenalidomide treatment. Also the percentage of CD33^+^ CD11b^+^ cells within the PBMC population showed a significant drop (29.86% versus 21.73%) during lenalidomide therapy.

Given the significant impact of lenalidomide maintenance on Treg numbers, we investigated further whether lenalidomide maintenance also affected the Treg phenotype. We measured the expression of CD45RA, CD28, T-cell immunoglobulin and mucin-domain containing-3 (TIM-3), T-cell immunoglobulin and immunoreceptor tyrosine-based inhibitory motif (TIGIT), lymphocyte activation gene 3 (LAG-3), CD27 and inducible T cell co-stimulator (ICOS) on Tregs by flow cytometry (Figure 2C). Of these markers, a significant increase in the percentage of CCR7^+^ (mean: 12.12 versus 18.29), CD62L^+^ (mean: 14,10 versus 28,69) and HLA-DR^+^ (mean: 25.75 versus 44.42) Tregs was observed upon lenalidomide treatment concomitant with a reduced percentate of CD45RA^+^ Tregs (40.37% versus 28.27%)

### Effects of lenalidomide maintenance therapy on T-cell expression of co-stimulatory and co-inhibitory molecules

**Figure 3:**
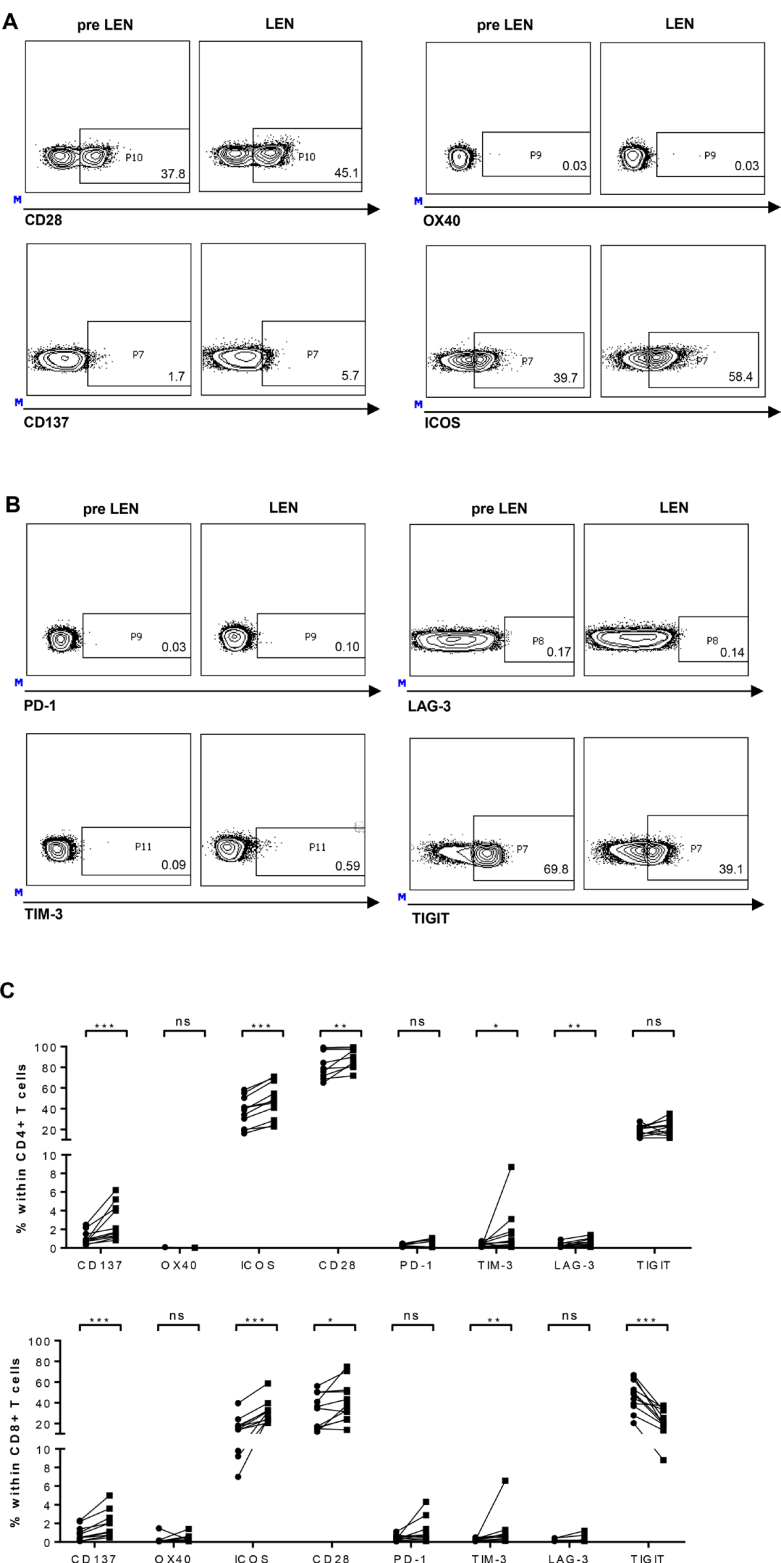
Effects of lenalidomide maintenance therapy on T-cell expression of co-stimulatory and co-inhibitory molecules PBMCs obtained before (pre LEN) and during (LEN) lenalidomide maintenance treatment were examined by flow cytometry to determine the expression of co-stimulatory and -inhibitory immune checkpoints (*n* = 11). The expression of co-stimulatory and -inhibitory immune checkpoints on CD8^+^ T cells of one patient are shown as example in panels (**A** and **B**), respectively. Numbers shown on the flow cytometry plots represent percentages of CD8^+^ T cells positive for the indicated immune checkpoint molecule. Overview graphs showing co-stimulatory and inhibitory -immune checkpoint expression on CD4^+^ and CD8^+^ T cells are shown in panel (**C**). The Wilcoxon matched-pairs signed rank test was performed to evaluate statistical significant differences, ns: not significant, ^*^p ≤ 0.05, ^**^p ≤ 0.01 and ^***^ *p* < 0.001.

The important role of stimulatory and inhibitory immune checkpoint molecules on the T-cell surface in tumor immunology is becoming more and more obvious. We investigated the expression of the co-stimulatory molecules CD28, CD137/4-1BB, OX40 and ICOS on the surface of CD4^+^ and CD8^+^ T cells. OX40 was the only of the co-stimulatory receptor of which the expression was not significantly up-regulated on the T cells during lenalidomide treatment (Figure 3, panels A and C). CD28 expression was observed on average on 31.30% and 41.41% of the CD8^+^ T cells before and during lenalidomide, respectively. For the CD4^+^ T cells the mean percentage of CD28^+^ cells raised from 85.15% to 90.65% during treatment. The mean percentage of CD137^+^ T cells raised from 0.89% to 1.85% and from 1.04% to 2.68% for the CD8^+^ and the CD4^+^ T-cell population, respectively. Finally, on average 16.59% CD8^+^ T cells expressed ICOS before lenalidomide treatment and this percentage increased to 31.19% during treatment. For the CD4^+^ T cells, on average 36.76% and 47.37% of the cells expressed ICOS before and during lenalidomide treatment, respectively.

For the co-inhibitory molecules, we observed a low (always <10% and typically <2%) expression of PD-1, TIM-3 and LAG-3 on T cells from MM patients, while the expression of TIGIT on both CD4^+^ and CD8^+^ T-cell compartments was much more pronounced (Figure 3, panels B and C). On the CD8^+^ T cells, the expression of TIM-3 was significantly increased upon lenalidomide maintenance therapy (mean: 0.26% versus 1.14%) while the percentage of TIGIT^+^ cells showed a significant drop (46.75% versus 23.97%). No significant effects were observed concerning PD-1 or LAG-3 expression on CD8^+^ T cells. On the CD4^+^ T cells, TIM-3 and LAG-3 expression were higher during lenalidomide treatment (0.36% versus 1.64% and 0.28 versus 0.55, respectively) whereas lenalidomide had no significant effects on expression of PD-1 or TIGIT.

### Lenalidomide maintenance therapy results in an improved CD8^+^ T cell functionality, which is hampered by non-CD8^+^ T cells present in the periphery

**Figure 4:**
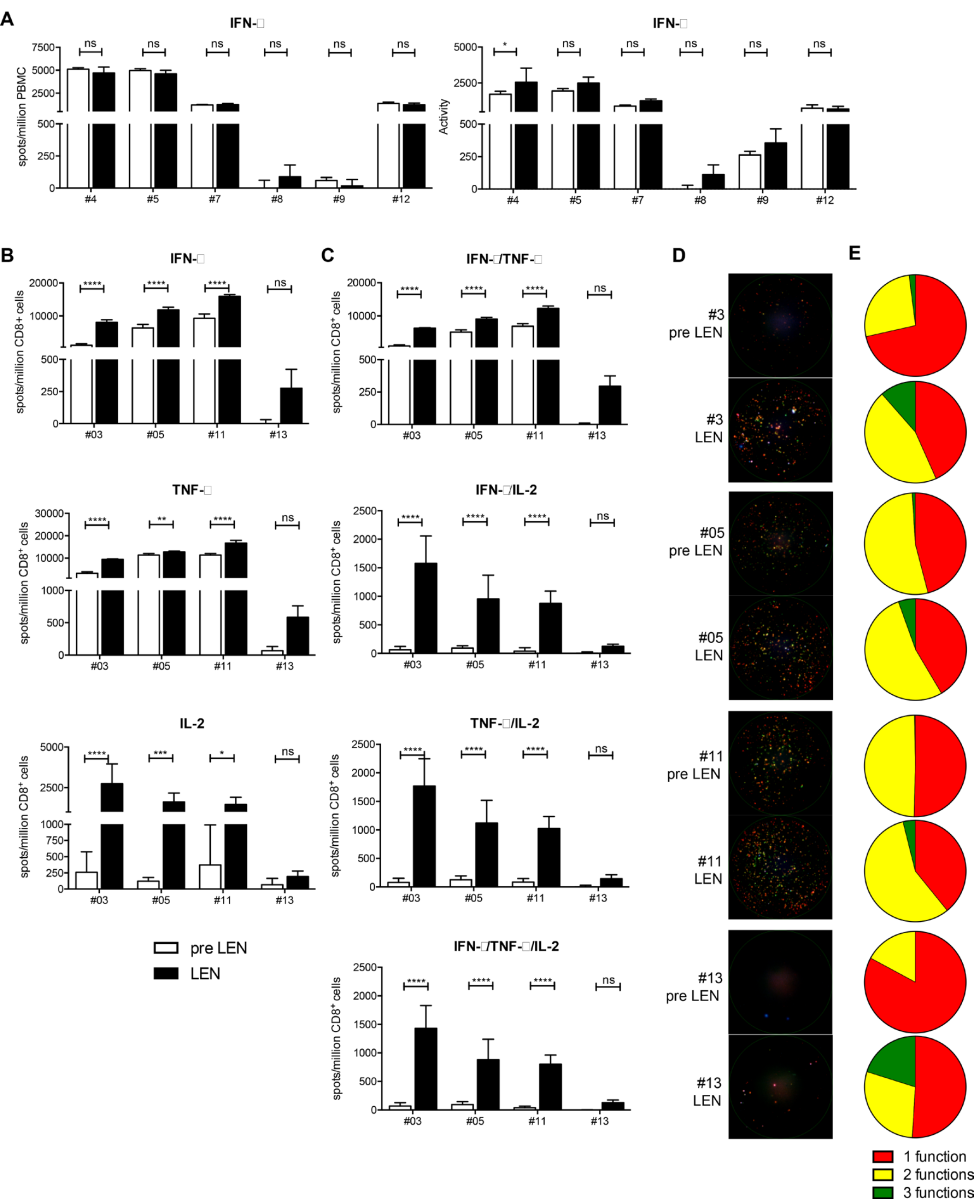
Lenalidomide maintenance therapy results in an improved CD8^+^ T cell functionality, which is hampered by non-CD8^+^ T cells present in the periphery (**A**) PBMCs obtained at the pre LEN and the LEN timepoint were examined by IFN-γ ELISPOT to measure the CD8^+^ T-cell response to viral recall epitopes derived for CMV, EBV and influenza (CEF) (*n* = 6). The number of spot forming units (SFU) as well as the activity in each well was determined. Bar graphs show the mean number of spots per million PBMCs or the mean activity of four replicate wells and the error bars show the standard deviation. Spot numbers and activity measured upon culture in the absence of peptide were subtracted. (**B**–**E**) CD8^+^ T cells purified from PBMC samples obtained at the pre LEN and LEN timepoint were examined by IFN-γ/IL-2/TNF-α FLUOROSPOT to characterize the CD8^+^ T-cell response to the CEF peptide pool. (B–C) Bar graphs show the mean number of spots per million CD8^+^ T cells of five replicate wells and the error bars show the standard deviation. Spot numbers and activity measured upon culture in the absence of peptide were subtracted. (**D**) Raw data showing three-color FLUOROSPOT results. One representative well is shown for each patient and timepoint. Cytokine-specific spots are shown in green (IFN-γ), red (TNF-α), blue (IL-2) or overlays of these colors. (**E**) The pie charts indicate the portion of mono- and polyfunctional CEF-specific CD8^+^ T cells. A two-way ANOVA with Sidak’s multiple comparisons test was performed to detect differences between the pre LEN and the LEN timepoint, ns: not significant, ^*^p < 0.05, ^**^p < 0.01, ^***^p < 0.001 and ^****^p < 0.0001.

A pool of peptides containing HLA-class I restricted epitopes from CMV, EBV and influenza (CEF) was used to stimulate PBMCs obtained before and during lenalidomide maintenance treatment. These peptides elicit recall responses in the majority of individuals expressing these common MHC class I alleles since the majority has been previously exposed to these pathogens. We measured the T-cell IFN-γ production with an *ex vivo* ELISPOT assay (Figure 4A). In 5 out of 6 patients, we could detect CEF-specific T cells before and during lenalidomide treatment. We did not observe an effect of the lenalidomide treatment on the number of spot forming units (SFU) (Figure 4A, left part). However, in 5 out of 6 patients, we visually observed larger and more intense spots. This was evidenced by a modest increase of the activity, which is a measure for the amount of cytokine that is released by the T cells (Figure 4A, right part). However the increased of the activity did only reach statistical significance in one patient (*p* = 0.0625).

To further elaborate on CD8^+^ T-cell functionality, we performed a three-parameter FLUOROSPOT assay, this time using purified CD8^+^ T cells stimulated with the same CEF peptide pool. Performing this assay, we observed a significant increased number of IFN-γ, TNF-α and IL-2 spots (Figure 4B). In addition, the degree of CD8^+^ T-cell polyfunctionality was significantly increased upon lenalidomide maintenance treatment (Figure 4, panels C-E). On average, the number of mono-functional spots (only IFN-γ, TNF-α or IL-2) increased 1.9 times upon lenalidomide maintenance treatment while the number of bi-functional spots (IFN-γ+TNF-α, IFN-γ+IL-2 or TNF-α+IL-2) increased 4.3 times and the number of three-functional spots (IFN-γ+TNF-α+IL-2) increased >17.6 times.

### Lenalidomide maintenance therapy does not enhance MM-specific CD4^+^ T-cell responses

**Figure 5:**
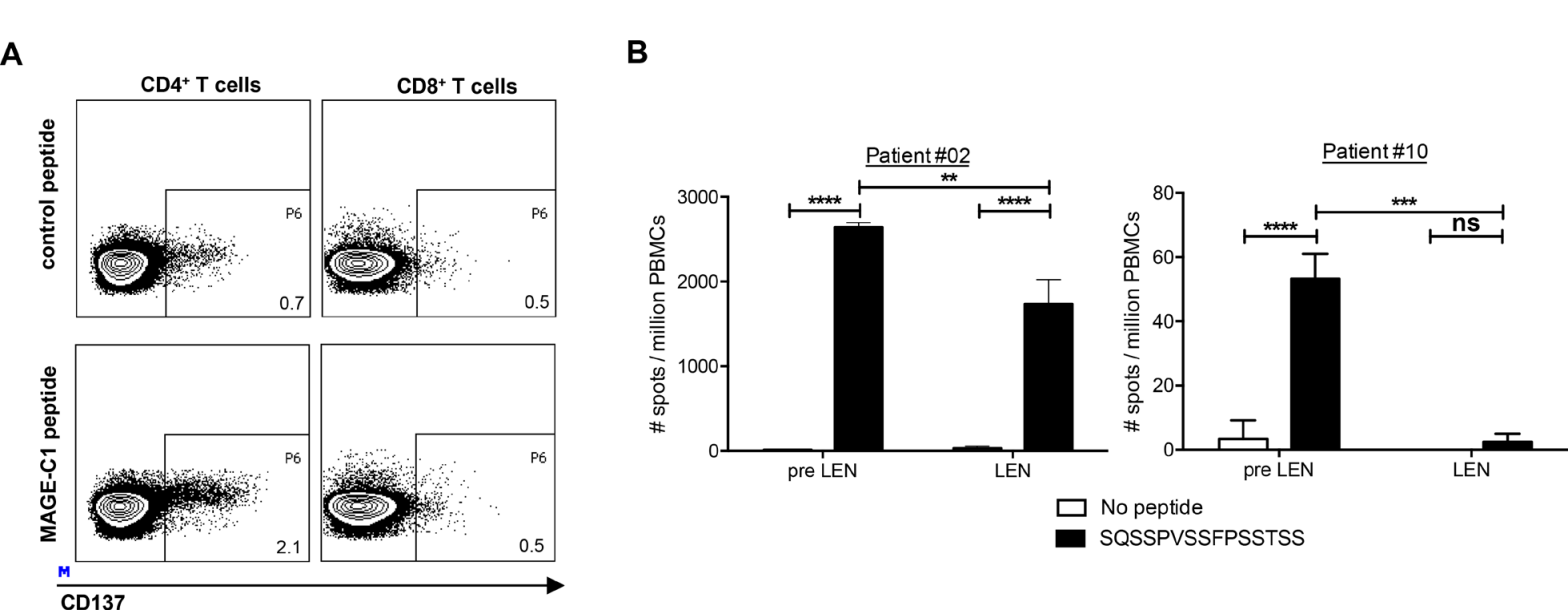
Lenalidomide maintenance therapy does not enhance MM-specific CD4^+^ T-cell responses Analysis of T-cell responses specific for the MAGE-C1 peptide SQSSPVSSFPSSTSS. (**A**) CD137 expression on CD4^+^ and CD8^+^ T cells from patient#02 at the pre LEN timepoint upon *in vitro* restimulation with the MAGE-C1 peptide. (**B**) PBMCs obtained at the pre LEN and the LEN timepoint were examined by IFN-γ ELISPOT to measure the T-cell response to the MAGE-C1 peptide. The bar graphs show the mean number of spots per million cells of four replicate wells and the error bars represent the standard deviation. A two-way ANOVA with Sidak’s multiple comparisons test was performed to evaluate statistical significant differences, ns: not significant, ^**^p ≤ 0.01, ^***^p ≤ 0.001 and ^****^p ≤ 0.0001.

Using cellular material from the pre LEN timepoint, we performed ELISPOT assays with peptide pools covering the MM-associated antigen MAGE-C1/CT7. Afterwards, individual peptides belonging to the peptide pools that were recognized in this first ELISPOT assay were tested in a second ELISPOT assay (data not shown). Remarkably, in two patients we detected reactivity against the same peptide (SQSSPVSSFPSSTSS). To our knowledge, this MAGE-C1 peptide was not previously described to be immunogenic. We performed a CD137 staining after stimulation with this peptide and observed that it was recognized by CD4^+^ T cells (Figure 5A). We compared the CD4^+^ T-cell responses specific for this MAGE-C1 peptide at both the pre LEN and the LEN timepoint in both patients (Figure 5B). In patient #02, the peptide was recognized at both timepoints, but the number of IFN-γ spots was higher at the pre LEN timepoint. In contrast to patient #02, patient #10 showed a very low pre LEN response to the peptide (53 spots/million cells) and we were not able to detect this response using PBMCs from the LEN timepoint. We did not find effects of the lenalidomide treatment on the MAGE-C1 specific ELISPOT activity.

## DISCUSSION

The positive effect of lenalidomide maintenance on PFS and survival after ASCT is generally ascribed to immune modulation of the myeloma microenvironment. A more profound understanding of the lenalidomide-induced changes in the immune environment in MM patients may provide new clues for immunotherapeutic interventions such as anti-tumor vaccination or checkpoint inhibition. We therefore performed a detailed analysis of the immune modulatory effects of lenalidomide maintenance therapy after ASCT in a homogeneously treated cohort of newly diagnosed MM patients. We only studied patients achieving a low tumor burden to minimalize immune effects of residual tumor cells. Also, such patients are likely to be most eligible for immunotherapeutic intervention as they benefit from a prolonged PFS.

Our major finding is that during lenalidomide maintenance a marked increase in the number of Tregs with mainly suppressive phenotype was observed. The existing data on Treg numbers in monoclonal gammopathies are not consistent. In patients with monoclonal gammopathy of unknown significance (MGUS) or MM, increased numbers of Tregs in the peripheral blood were found, associated with MM progression [17–22]. In contrast, two studies actually showed reduced numbers of Tregs in MGUS and MM patients [23, 24]. In more advanced myeloma, lenalidomide has been shown to increase the number of circulating Treg [14, 25–28]. We and others previously showed that Tregs from MM patients are endowed with a potent immune suppressive capacity [5, 17, 19, 24, 29]. Some studies however have shown that these Treg have diminished immunosuppressive functional capacity [23, 30]. Conflicting results regarding Treg numbers and suppressive capacity are most likely in part explained by different flow cytometry gating strategies and patient’s heterogeneity regarding disease stage or treatment. To assess Treg functionality in this study we evaluated the Treg phenotype and found that the expression of HLA-DR, CCR7 and CD62L was increased while CD45RA was reduced during lenalidomide maintenance. HLA-DR^+^ Tregs are characterized by contact-dependent immune suppression and a high Foxp3 expression [31]. CCR7 expression on naive Tregs enables them to enter the lymph node to control the priming phase of an immune response while CCR7 expression on effector/memory like Tregs supports emigration from the inflamed site, resulting in a more efficient immune suppression [32]. CD62L^+^ Tregs have a high suppressive and proliferative potential [33]. CD45RA^–^ Tregs are so-called activated Tregs, endowed with a strong suppressive function and a high proliferative capacity [19]. Taken together, our data clearly demonstrate that lenalidomide maintenance actually enhances the suppressive function of the Treg compartment.

Regarding the effector T-cell compartment, we observed increased naive CD8^+^ T cells and memory CD4^+^ and CD8^+^ T cells whereas terminal effector T cells decreased. These terminal effector T cells are characterized by a reduced proliferative potential and a decreased multipotency [16]. Furthermore, we found that the CD8+ T cells obtained after lenalidomide treatment produced higher amounts of the pro-inflammatory cytokines IFN-γ and TNF-α and of the chemokine MIP-1β upon polyclonal stimulation. The CD4^+^/CD8^+^ T cell ratio, known to decrease at MM progression [34, 35], was increased. These findings support previous observations showing the immune-activating effects of lenalidomide in MM patients [11].

Since memory T-cells may play an important role within the context of prolonged lenalidomide maintenance treatment we also examined percentages and functionality of these cells. When we stimulated whole PBMC, we did not find an effect of the lenalidomide treatment on the number of CEF-specific CD8^+^ T cells. However, when we performed a FLUOROSPOT assay using purified CD8^+^ T cells, we found significantly increased percentages of antigen-specific CD8^+^ T cells with a more polyfunctional profile. Polyfunctional T cells may have more anti-myeloma potential. Given the significantly increased percentages of Tregs with a more suppressive phenotype upon during lenalidomide maintenance treatment, we hypothesize that the Tregs present in the PBMC samples might have hindered the CD8^+^ T-cell costimulatory effects of lenalidomide in the first assay. Some studies have investigated antigen specific T-cell responses upon treatment with a combination of lenalidomide and vaccination. Noonan *et al.* investigated vaccination with pneumococcal 7-valent conjugate vaccine (PCV). MM patients who received the PCV vaccine while being treated with lenalidomide showed increased numbers of MM-specific IFN-γ producing T cells in the bone marrow [36]. In addition, Krämer *et al.* showed an increased frequency of T cells specific for the MM antigen HM1.24 in patients receiving lenalidomide maintenance treatment [14]. MAGE-C1, a well-studied tumor associated antigen, is expressed on myeloma cells and may be a potential target for immunotherapy [37]. However, we did not observe an effect of lenalidomide maintenance on the frequency of MAGE-C1 specific CD4^+^ T cells in our patients.

The expression of inhibitory immune checkpoint molecules in the MM microenvironment is of particular interest in view of the exciting results currently observed with the use of checkpoint inhibitors in various neoplastic diseases. High percentages of PD-1^+^ CD4^+^ and CD8^+^ T cells are observed in MM patients [6, 38], but following ASCT, T-cell expression of PD-1 returns to levels seen in healthy individuals [38]. Also bone marrow cytotoxic T cells from MM patients express only low levels of PD-1 [39]. These finding may explain why phase I clinical studies have failed to show significant clinical responses of PD-1 blockade in MM [39].

Our analysis of immune checkpoint molecules during lenalidomide maintenance therapy most strikingly showed that T cells form MM patients post ASCT express high levels of TIGIT, which was significantly higher than its expression on T cells from healthy individuals (data not shown). TIGIT is a recently identified inhibitory immune checkpoint receptor that is expressed on activated T cells, Tregs and NK cells. Interestingly, high frequencies of TIGIT^+^ CD8^+^ T cells are also detected in patients with acute myeloid leukemia (AML) and are associated with poor clinical outcome. In AML patients, TIGIT^+^ CD8^+^ T cells exhibit features of exhaustion [40]. Lenalidomide maintenance decreased the number of TIGIT^+^ CD8^+^ T cells significantly, yet increased TIM-3^+^ and LAG-3^+^ CD4^+^ T cells and TIM-3^+^ CD8^+^ T cells were observed. However, the overall expression of TIM-3 and LAG-3 was relatively low. We also observed an increased expression of the immune stimulatory antigens CD137, ICOS and CD28 within both the CD4^+^ and the CD8^+^ T-cell populations, with no effect on the OX40 expression.

We previously showed the suppressive capacity of the CD11b^+^ CD14^+^ HLA-DR^low^ monocytic MDSC population *in vitro* [5]. We found a modest, but significant, reduction in the percentages of CD14^+^ HLA-DR^low^ PBMCs and CD11b^+^ CD33^+^ cells during lenalidomide maintenance treatment. In contrast, a previous study showed that patients treated with lenalidomide have high numbers of CD14^+^ CD15^+^ myeloid cells able to inhibit T-cell proliferation *in vitro* [11] At this moment, there is no consensus on the frequency and function of moMDSCs in MM patients. Görgün *et al.* found that CD11b^+^ CD14^+^ HLA-DR^–/low^ MDSCs demonstrated to be either absent or less immune suppressive compared to the CD11b^+^ CD14^–^ HLA-DR^–/low^ CD33^+^ CD15^+^ MDSCs in the MM micro-environment [41]. Brimnes *et al.* showed increased numbers of CD14^+^ HLA-DR^–/low^ MDSCs in MM patients at diagnosis [29]. However, Ramachandran *et al.* demonstrated that although there is no difference in the number of CD11b^+^ CD14^+^ HLA-DR^–/low^ cells between MM patients and healthy individuals, these moMDSCs isolated from the BM exert a suppressive function in MM patients but not in healthy individuals [42]. Recently, Wang *et al.* showed that increased frequencies of moMDSCs are associated with MM tumor progression [43]. Granulocytic (CD15^+^) MDSCs are depleted from the PBMCs and could not be investigated in our study.

After the LEN timepoint, the patients in this study underwent vaccination with autologous DCs electroporated with the MM-associated antigens MAGE-C1 and MAGE-A3 while continuing lenalidomide maintenance (EUDRACT 2013-000795-15). Clinical and immunological responses to the vaccine are investigated thoroughly and will further clarify the impact of lenalidomide maintenance treatment on the efficacy of the vaccination.

In summary, our data show that lenalidomide maintenance *in vivo* induces 1) increased percentages and suppressive phenotype of Tregs 2) T cell activation and CD8^+^ T cell antigen specific responses 3) more expression of T-cell co-stimulatory but also increased expression of some T-cell co-inhibitory molecules, except for TIGIT 4) diminished activity of moMDSCs. In view of the positive effects of lenalidomide maintenance on PFS and survival in myeloma patients after ASCT the balance of these effects must be in favor of immune-stimulation against residual myeloma cells. However it is likely that additional immune interventions such as anti-tumor vaccinations could be hampered, especially by Treg expansion. Interventions aimed at reducing Tregs during lenalidomide maintenance such as the use of cyclophosphamide or antibodies such as anti-CD38 may be needed in an attempt to make these interventions successful.

## MATERIALS AND METHODS

### Study subjects

Newly diagnosed, transplant eligible MM patients (aged ≤ 65) years were recruited from 3 Belgian University Hematology departments: Universitair Ziekenhuis Brussel (Brussels), Centre Hospitalier Universitaire de Liège (Liège) and Institut Jules Bordet (Brussels). Approval for this study was obtained from the institutional review boards and informed consent was obtained from all participating patients prior to their inclusion in this study, according to the Declaration of Helsinki. This study is registered in the European Clinical Trials Database (EUDRACT Study Number: 2013-000795-15). Response assessment was done according to the IMWG response criteria [44]. Patients were eligible for study participation if they obtained a very good partial response (VGPR) or better either after single ASCT or 2 additional cycles of a bortezomib based consolidation. Response assessment was performed at 100 days after ASCT or after the end of consolidation. Maintenance therapy consisted of low dose lenalidomide (10 mg qd) continuously until progression.

### Cellular material

Peripheral blood mononuclear cells (PBMCs) were isolated from leukapheresis (pre-LEN timepoint) and whole blood (LEN timepoint) samples through lymphoprep gradient-centrifugation (Axis-Shield) following the manufacturer’s instructions before being cryopreserved.

### Flow cytometry

Flow cytometry analyses were performed on an LSR Fortessa flow cytometer (BD Biosciences). Automatic compensation was performed using CompBeads (BD Biosciences). Fluorescence minus one controls were performed to define gates of positivity. Following antibodies and reagents were used: biotinylated anti-CD11b, anti-CD33 PE-Cy7, anti-HLA-DR PE-Cy7, anti-CD15 FITC, anti-CD8 PE, anti-CD4 APC-Hy, anti-CD127 FITC, anti-CD16 FITC, anti-CD45 RO PE, andi-CD45 RA FITC, anti-CD95 PD-CF594, anti-PD-1 APC, anti CD62L APC (BD Biosciences); anti-CD14 APC-Cy7, anti-CD3 BV 605, anti-CD3 PE/Dazzle 594, anti-CD4 PE-Cy7, anti-CD4 PerCPCy5.5, anti-CD56 PE-Cy7, andi-CD28 FITC, anti-CD27 APC, anti-ICOS APC-Cy7, anti-CD137 PE, anti-CD137 APC, Zombie Yellow Fixable Viability Kit (BioLegend); eFluor 450 labeled streptavidin, anti-CD8 APC-H7, anti-Foxp3 APC, anti-CCR7 APC, anti-TIM3 eFluor 450, anti-TIGIT PE, anti-LAG3 PerCPeFluor710 (eBioscience), anti-CD25 PE (Myltenyi Biotec) and anti-OX40 APC (R&D Systems).

### Multiplex assay

CD4^+^ T cells and CD8^+^ T cells were purified using CD4 or CD8 MicroBeads (MACS technology, Miltenyi Biotec), respectively and stimulated Human T-activator CD3/CD28 dynabeads (Invitrogen) for 24 hours. Supernatants were collected and analyzed using theBio-Plex Pro Human Cytokine 17-plex (Bio-rad).

### Peptides

CEF-Class I Peptide Pool “Plus” was obtained from CTL. MAGE-C1/CT7 peptides with a length of 15 amino acids and with 11 amino acids overlap were obtained from EMC Microcollections. Peptides were stored in aliquots at –20° C.

### ELISPOT assays

The human IFN-γ ELISPOT set from Diaclone was used, in combination with PVDF ELISPOT plates (Millipore), as previously described [45]. ELISPOT Plates were evaluated using the AID ELISPOT automated reader system (Autoimmun Diagnostika GmbH) with accessory software.

### *Ex vivo* CEF ELISPOT

PBMCs were thawed, rested for 2 hours at 37° C, 5% CO2 in a humidified incubator. Afterwards, the cells were plated in the ELISPOT plates together with following stimuli: medium only (as a negative control) or 0.4 μg/ml CEF peptide pool.

### Detection of MAGE-C1 specific T cells by ELISPOT upon *in vitro* restimulation

MAGE-A3 and MAGE-C1-specific T cells were expanded prior to the IFN-γ ELISPOT as described previously [46]. The following stimuli were used to screen for antigen-specific T cells in the ELISPOT: medium only (as a negative control), anti-CD3 and anti-CD28 coated microbeads (Invitrogen, as a positive control) or the peptide used for the *in vitro* expansion (1 μg/ml).

### FLUOROSPOT assays

CD8^+^ T cells were purified using CD8 MicroBeads (MACS technology, Miltenyi Biotec) and plated in the pre-coated IFN-γ/IL-2/TNF-α FLUOROSPOT plates (Mabtech) together with following stimuli: medium only (as a negative control) or 0.4 μg/ml CEF peptide pool. The FLUOROSPOT plates were developed according to the manufacturers’ instructions and evaluated using the ImmunoSpot S6 Ultra-V (CTL) with accessory software.

### Data analysis

Flow cytometry data were analyzed using FACS DIVA (BD Biosciences) and FlowJo (Tree Star inc.) software. Statistical analysis was performed using GraphPad Prism software.

## SUPPLEMENTARY MATERIALS FIGURES


