# Supplementary material for: Impact of lenalidomide maintenance on the immune environment of multiple myeloma patients with low tumor burden after autologous stem cell transplantation
Source: Oncotarget. 2018 Apr 17;9(29):20476–89. doi: 10.18632/oncotarget.24944 (PMC5945510; doi:10.18632/oncotarget.24944)
Supplement: Supplementary file 1 [file oncotarget-09-20476-s001.pdf]

Impact of lenalidomide maintenance on the immune environment of multiple myeloma patients with low tumor burden after autologous stem cell transplantation

SUPPLEMENTARY MATERIALS

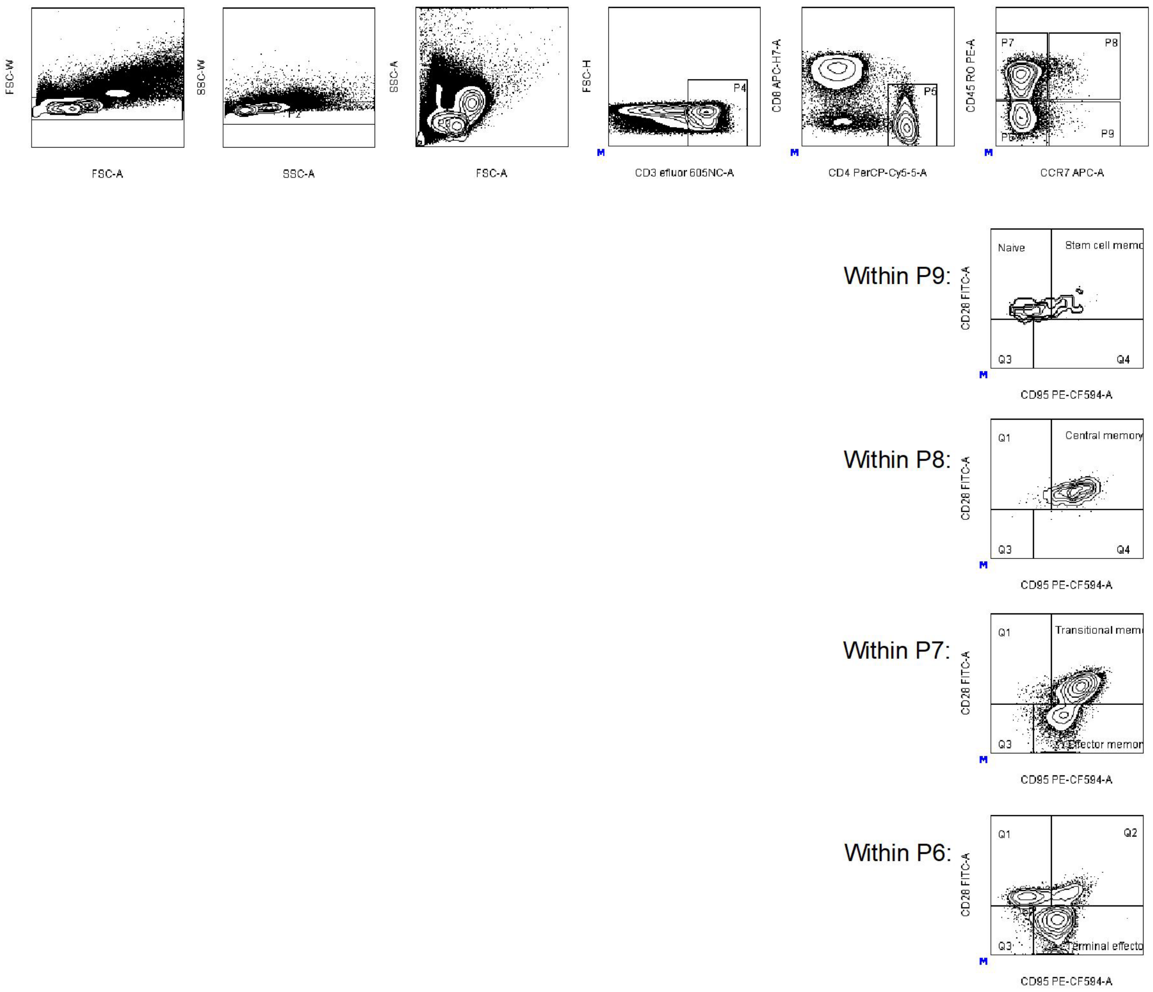

Supplementary Figure 1: The flow cytometry plots show the T cell subset gating strategy. First, we gated on single cells using FSC-A/FSC-W and SSC-A/SSC-W plots. Next, we gated on the lymphocytes using FSC-A/SSC-A plots. Within the $CD3^+$ population, we gated on $CD4^+$ and $CD8^+$ T cells. Next, we identified 4 populations based on CCR7 and CD45RO expression. Within $CD45RO^-$ $CCR7^+$ population (P9): naïve ($CD28^+$ $CD95^-$) and stem cell memory ($CD28^+$ $CD95^+$) T cells were identified. Within the $CD45RO^+$ $CCR7^+$ population (P8) the central memory T cells ($CD28^+$ $CD95^+$) T cells were identified. Within the $CD45RO^-$ $CCR7^+$ population (P7), the transitional memory ($CD28^+$ $CD95^+$) and the effector memory T cells ($CD28^-$ $CD95^+$) were identified. Finally, within the $CD45RO^-$ $CCR7^-$ population (P6), the terminal effector T cells ($CD28^-$ $CD95^-$) were identified.

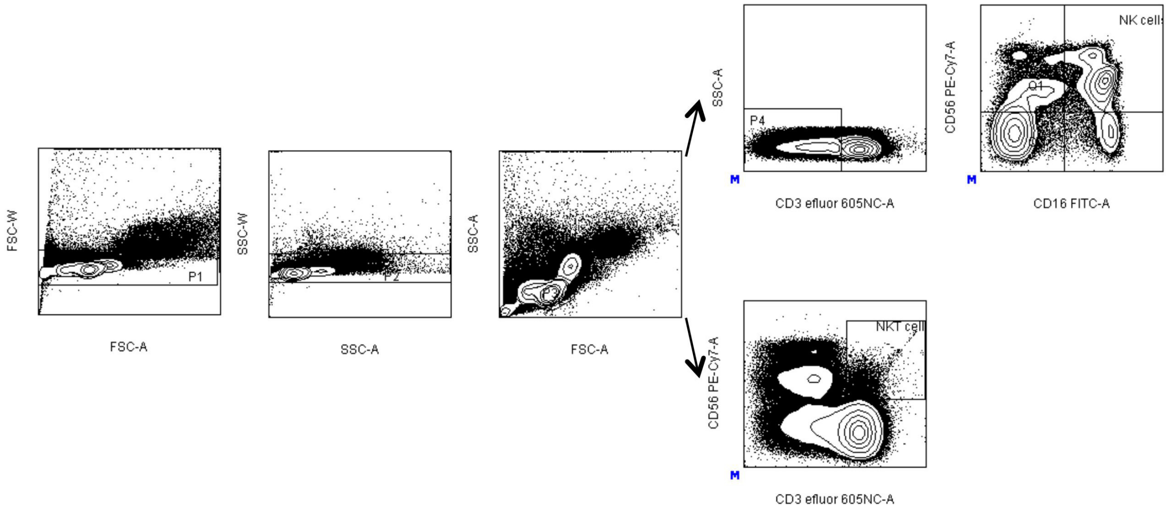

Supplementary Figure 2: The flow cytometry plots show the NK and NKT cell subset gating strategy. First, we gated on single cells using FSC-A/FSC-W and SSC-A/SSC-W plots. For the NK cells, we first gated on CD3- cells. Within the $CD3^-$ population, NK cells were defined as $CD56^+$ $CD16^+$ cells. NKT cells were defined as $CD3^+$ $CD56^+$ cells.

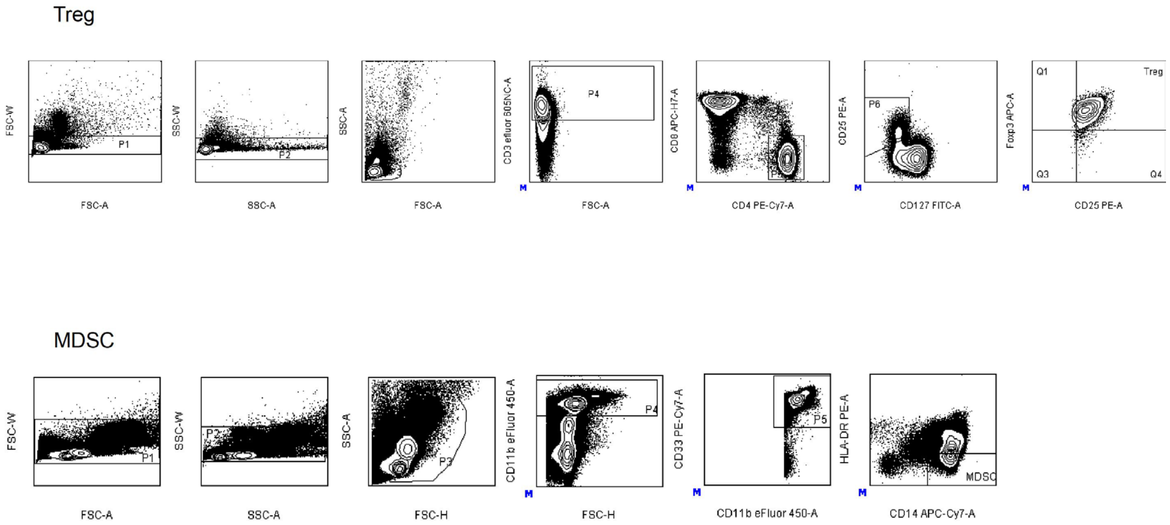

Supplementary Figure 3: The flow cytometry plots show the gating strategy for immune suppressive cell types. For the Treg stainings, we first gated on single cells using FSC-A/FSC-W and SSC-A/SSC-W plots. Next, we gated on the lymphocytes using FSC-A/SSC-A plots. Within the $CD3^+$ population, we gated on $CD4^+$ T cells. Next, we gated on the $CD25^{high}$ $CD127^{low}$ population (P6). Within P6, we gated on the $Foxp3^{high}$ cells. For the MDSC stainings, we first gated on single cells using FSC-A/FSC-W and SSC-A/SSC-W plots. Next, we gated on the viable cells using FSC-A/SSC-A plots. Within the $CD11b^+$ $CD33^+$ population, we gated on $CD14^+$ HLA-DR^{low} cells.

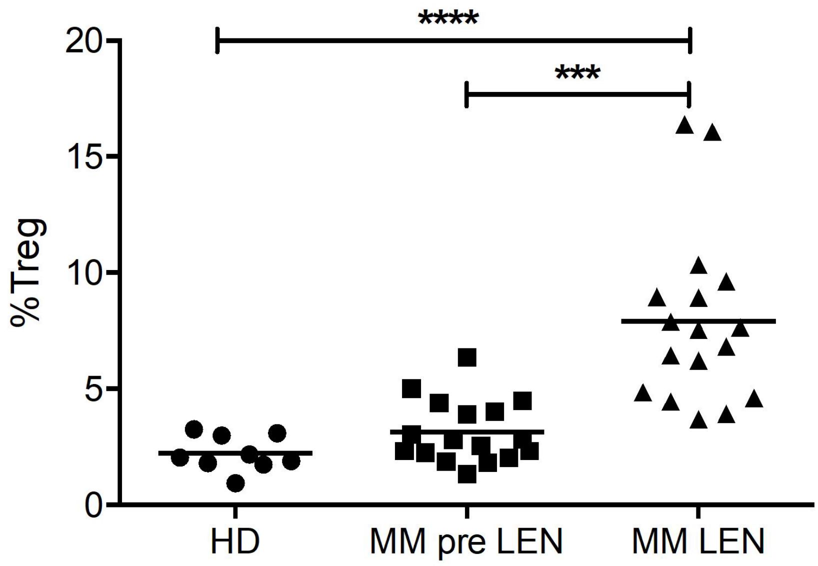

Supplementary Figure 4: The graphs shows the Treg frequency in healthy donors compared to MM patients before or during lenalidomide maintenance therapy. The Kruskall–Wallis test was performed to evaluate statistical significant differences, $^{***}p < 0.001$, and $^{****}p < 0.0001$.
